# The Effectiveness of Transversus Abdominis Plane and Quadratus Lumborum Blocks in Acute Postoperative Pain Following Cesarean Section—A Randomized, Single-Blind, Controlled Trial

**DOI:** 10.3390/ijerph18137034

**Published:** 2021-06-30

**Authors:** Michał Borys, Beata Potręć-Studzińska, Paweł Kutnik, Justyna Sysiak-Sławecka, Elżbieta Rypulak, Tomasz Gęca, Anna Kwaśniewska, Mirosław Czuczwar, Paweł Piwowarczyk

**Affiliations:** 1 II Department of Anesthesiology and Intensive Care, Medical University of Lublin, Ul. Staszica 16, 20-081 Lublin, Poland; beatapotrec@gmail.com (B.P.-S.); 50159@student.umlub.pl (P.K.); justynasysiak@wp.pl (J.S.-S.); elzbieta.rypulak@umlub.pl (E.R.); czuczwarm@gmail.com (M.C.); pawelpiwowarczyk@umlub.pl (P.P.); 2 Department of Obstetrics and Pathology of Pregnancy, Medical University of Lublin, 20-081 Lublin, Poland; tomasz.geca@umlub.pl (T.G.); anna.kwasniewska@umlub.pl (A.K.)

**Keywords:** pain, cesarean section, analgesia, quadratus abdominis block, transverse abdominis plane block

## Abstract

Acute pain intensity related to cesarean section (CS) may be extensive and is often underestimated. This may influence mothers’ quality of life and their children’s development. Regional analgesia techniques that include transversus abdominis plane block (TAPB) and quadratus lumborum block (QLB) have proven their efficacy in the postoperative period after CS. Although several randomized controlled studies and one meta-analysis have investigated the utility of TAPB and QLB in the reduction of acute and chronic pain after CS, only one study directly compared both types of regional blocks and revealed superiority of QLB over TAPB. Our study aimed to reevaluate the effectiveness of transversus TAPB and QLB in controlling acute postoperative pain after CS. We recruited 197 women with singleton pregnancies undergoing CS under spinal anesthesia. The patients were randomized to receive either TAPB or QLB after CS. The acute postoperative pain was evaluated using the visual analog scale (VAS) at 2, 4, 8, 12 and 24 h after the operation. No significant difference in acute postoperative pain intensity between the groups was found. The patients who received TAPB had a higher demand for supplemental morphine injections (*p* < 0.039). In our study, none of the evaluated regional blocks demonstrated an advantage over the other regarding acute postoperative pain management.

## 1. Introduction

The number of the cesarean deliveries has almost doubled between 2000 and 2015 [1]. The highest rise of cesarean sections (CSs) in recent years was observed in Eastern Europe and South and Central Asia. In Poland, the percentage of childbirth by cesarean section reached 38.9% of all deliveries in 2018 [2]. Acute pain intensity related to this procedure is often underestimated. Gerbershagen et al. noted that of 179 types of surgical procedures that were performed in Germany, the cesarean delivery was graded as the ninth in terms of postoperative pain intensity [3]. The authors concluded that ineffective pain treatment after CS in comparison with large abdominal/thoracic procedures may be attributed to the use of regional anesthesia techniques, especially epidural in the latter.

Moreover, Marcus et al. revealed that 63% of German parturients did not receive opioids in the postoperative period [4]. Several factors can affect the use of opioids in this specific population, such as respiratory depression or excessive sedation in neonates [5]. Poorly treated postoperative pain may lead to compromised early mother–child interaction due to postpartum depression and consequently to deterioration of the child’s development [6,7,8].

The effectiveness of transversus abdominis plane block (TAPB) has been demonstrated as a part of multimodal analgesia after cesarean delivery; however, its action may be limited to somatic pain and apparently only when intrathecal morphine is not administered [9]. Rafael Blanco developed a novel type of truncal block, called a quadratus lumborum block (QLB), and showed its usefulness in parturients [10]. Recently, several randomized controlled studies and one meta-analysis have provided investigations into the utility of TAPB and QLB in the reduction of acute and chronic postoperative pain after CS [11,12,13,14,15,16]. However, only one study directly compared the two types of regional blocks, which revealed the superiority of QLB over TAPB [11].

Our study aimed to reassess the effectiveness of TAPB and QLB in acute postoperative pain management after cesarean section.

## 2. Material and Methods

### 2.1. Study Design

This prospective randomized single-blind trial was conducted in a tertiary obstetric department. The study protocol was approved (permit number KE-0254/85/2016) by the local bioethics committee of Medical University of Lublin, in Lublin, Poland (Prof. Olajossy), and was registered at ClinicalTrials.gov (NCT02804126). Informed, written consent was obtained from every patient, and the study methods were in line with the tenets of the Declaration of Helsinki for medical research involving human subjects.

We included pregnant females (singleton pregnancy) older than 18 years who were scheduled for cesarean section under single-shot spinal anesthesia.

We excluded patients with coagulopathy, allergies to local anesthetics, depression, antidepressant drug therapy, epilepsy, chronic painkiller use before surgery, addiction to alcohol or recreational drugs or gestational age below 36 weeks.

### 2.2. Anesthesia and Regional Block

**Figure 1:**
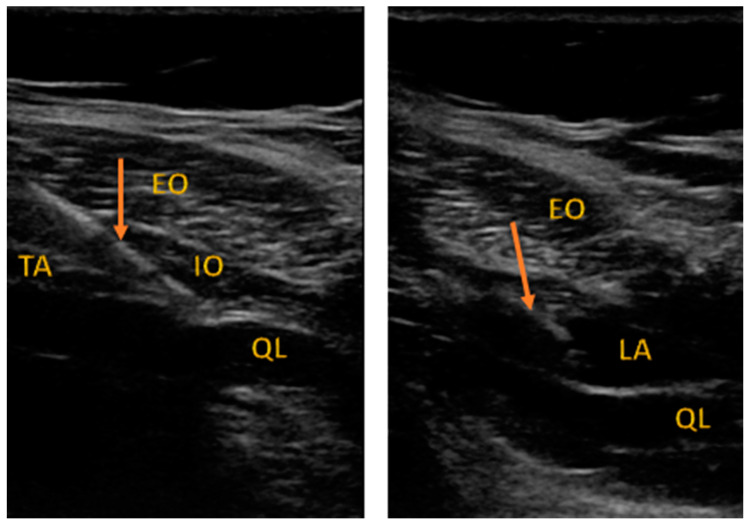
Sonoanatomy of QLB performed in the study.

**Figure 2:**
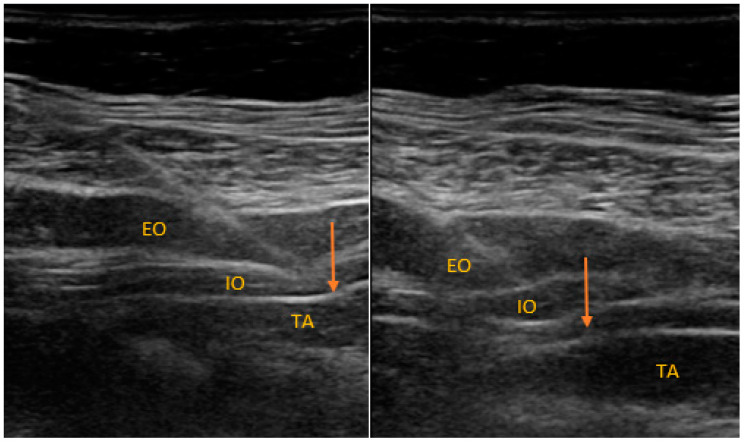
Sonoanatomy of TAPB performed in the study.

All the patients received single-shot spinal anesthesia with a 0.5% solution of hyperbaric bupivacaine. The patients received spinal injections from 2.0 to 3.0 mL of local anesthetic to reach a sufficient level of the blockade (T4–T6). Only individuals with an appropriate level of anesthesia could be randomized. At the end of the procedure, the participants were randomly allocated into one of the study arms (1:1 allocation ratio): TAPB or QLB, for ultrasound-guided regional blockade (Figure 1 and Figure 2). The team member who anesthetized the patient opened an opaque envelope that contained the patient’s study group allocation. The envelope was sealed, and the patient allocation was prepared according to the randomization procedure by a team member not directly involved in the process of anesthesia or further evaluation of the participants.

Figure 1 describes the approach to QLB type 2. EO: external oblique, IO: internal oblique, TA: transversus abdominis, QL: quadratus lumborum. The arrow indicates the needle shaft.

Figure 2 describes the approach to TAPB. EO: external oblique, IO: internal oblique, TA: transversus abdominis. The arrow indicates the needle shaft.

**Figure 3:**
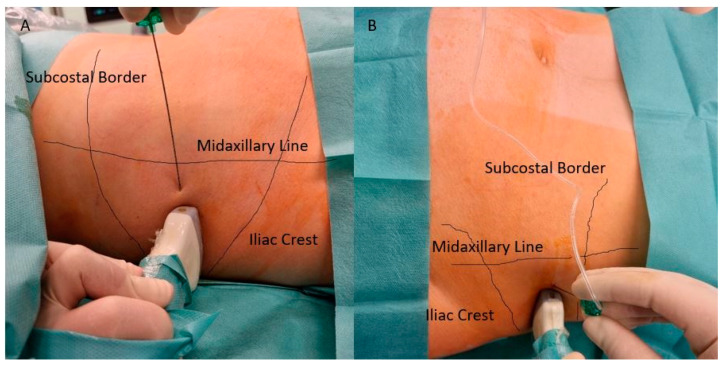
Site of administration of QLB and TAPB performed in the study. (**A**) Approach of administration of QLB; (**B**) Approach of administration of TAPB. Both blocks are performed between Illiac Crest and lower border of rib cage, below Midaxillary Line and guided under linear ultrasonography as presented in Figure 1 and Figure 2.

Figure 3 presents site of administration of QLB (A) and TAPB (B).

We chose the second type of QLB as described by Blanco [17]. However, we used a linear probe to identify an optimal point of injection, similarly to the description presented by Ueshima et al. [18]. According to the study protocol, the patients were not informed about the type of regional block performed on them (single-blind or computer-generated randomization). Only three physicians performed the regional blocks (MB, BPS and PP).

The block was administered bilaterally with a 0.25% solution of bupivacaine (from 0.2 mL of local anesthetic per kilogram to a maximum dose of 20 mL per side). Every block was performed in the operating theater before the transfer of patients to the ward.

### 2.3. Postoperative Pain Assessment

Postoperative pain intensity was measured using the visual analog scale (VAS) at the 2nd, 4th, 8th, 12th and 24th hour after the patients’ referral to the postoperative care unit. VAS forms were collected by midwives who were not aware of the block type. The standard postoperative pain management included two of four intravenously administered drugs: paracetamol, ketoprofen, diclofenac and tramadol. Each painkiller was administered at the scheduled time point. In the case of severe pain (more than 40 mm on VAS), up to two dosages of subcutaneous (s.c.) morphine (5 mg) were administered based on a midwife’s discretion.

### 2.4. Statistics

Analysis of variance (ANOVA) and Student’s *t*-test were used to analyze parametric data. Tukey’s honest significant difference test was used for post-hoc analysis. The results obtained using the VAS were presented as means and confidence intervals (CI). The statistics for nonparametric data were calculated using the Mann–Whitney U test or the Kruskal–Wallis test by ranks and were presented as medians and interquartile ranges. Fisher’s exact test was used to analyze the contingency tables. All the measurements were performed using Statistica 12.5 software (Stat Soft. Inc., Tulsa, OK, USA).

### 2.5. Power Analysis

Our sample size was calculated based on the results obtained by Blanco et al., where the use of two regional blocks in alleviating acute postoperative pain was compared [11]. Because we did not use the patient-controlled analgesia (PCA) technique, we calculated a sample size according to pain intensity measured on the VAS scale. We obtained the raw data from the study by Blanco on the web site of the “Regional Anesthesia and Pain Medicine” journal. We considered five measurements of pain severity, which were obtained during the first 24 h. The mean VAS result (five evaluations) in patients after the TAPB was 19 and 15 mm in the QLB group. The calculated sample size to obtain a significant difference was 200 participants, 100 per group (alfa 0.05, power 0.8).

### 2.6. Outcomes

The primary outcome of our study was to evaluate pain severity in patients after QLB and TAPB at the 2nd, 4th, 8th, 12th and 24th hour. The secondary outcomes included the consumption of antinociceptive drugs during the first postoperative day and the correlation between pain intensity and indications for the cesarean section.

## 3. Results

### 3.1. Patient Demographics

**Figure 4:**
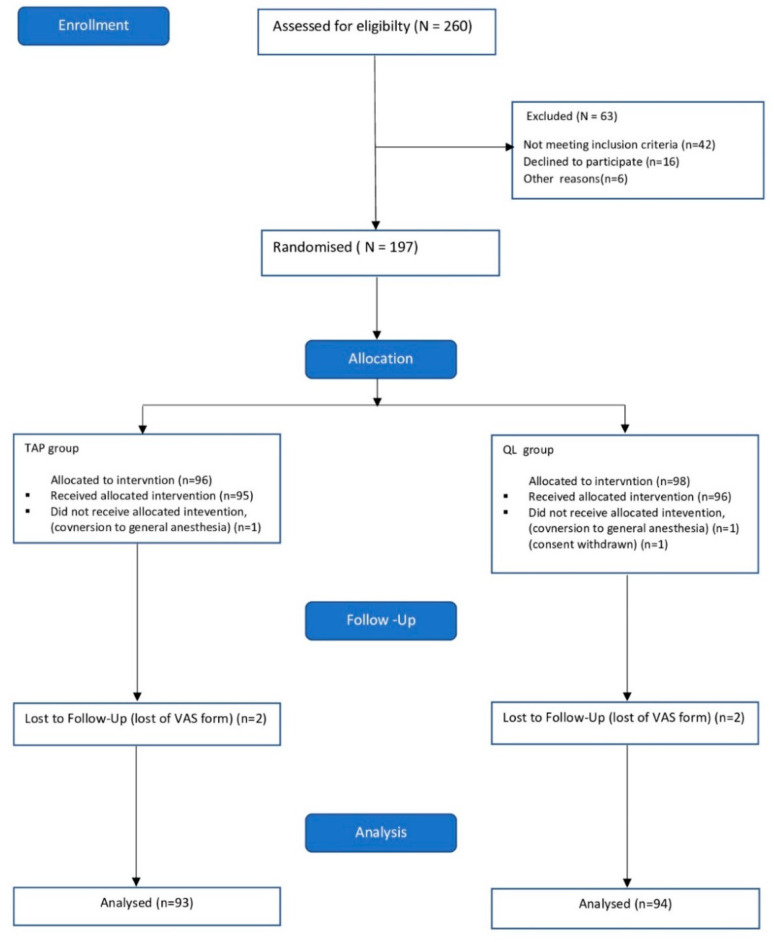
Study flowchart.

The study was conducted from June 2017 to February 2018. The CONSORT flowchart is presented in Figure 4. The patient demographics are shown in Table 1. No differences were found in age, weight, height and BMI between the study groups. The indications for cesarean deliveries are listed in Table 2.

### 3.2. Acute Postoperative Pain

No difference in pain severity was found on the first postoperative day between the study groups. The VAS results are presented in Table 3.

### 3.3. Antinociceptive Drugs

No statistical significance was found in the total postoperative analgesic consumption between the study groups (Table 4). However, more patients in the TAPB group required supplemental subcutaneous morphine injections when compared with the QLB group (60 versus 46 patients, *p* = 0.039).

### 3.4. Correlation between Pain Intensity and Indications for Cesarean Section

**Figure 5:**
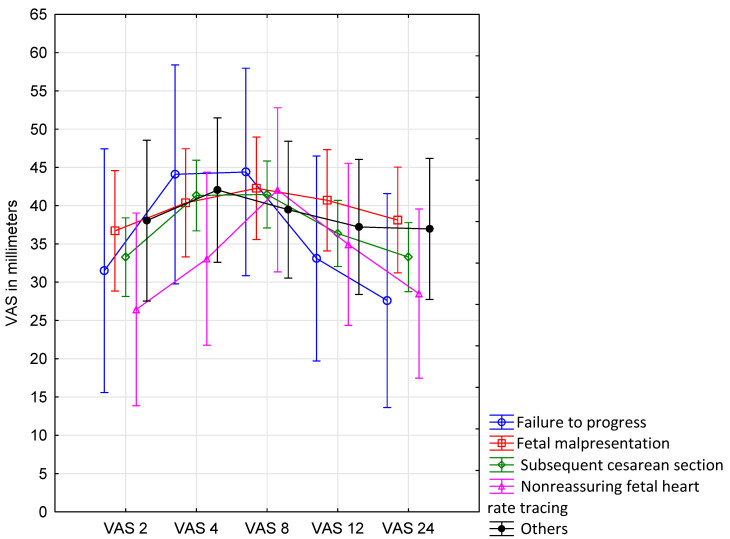
Postoperative pain severity during the first 24 h after the surgery according to indications for cesarean section. The plots represent VAS results according to the type of indication for cesarean section (without the division to the study groups). The results from each measurement are shown as a mean. Vertical bars denote 0.95 confidence intervals (CI).

The postoperative pain severity was not affected by the indication for cesarean section (Figure 5, Table 5).

## 4. Discussion

The primary goal of our study was to compare two techniques of regional anesthesia, TAPB and QLB, in their effectiveness in postoperative pain management in obstetric patients. However, we did not find a difference between the methods according to the primary outcome of our study. Moreover, no difference was found in the total postoperative analgesic consumption between the TAPB and QLB groups. The only significant difference demonstrated in our study was associated with a higher number of TAPB patients who received s.c. morphine in the postoperative period in comparison to QLB.

The number of techniques available to enhance postoperative pain control in puerperal patients is limited [19]. The preemptive administration of antiepileptic drugs, such as gabapentinoids, lidocaine and ketamine, is controversial due to the transfer to the baby through the placenta and breastfeeding. Therefore, the broader implementation of regional anesthesia techniques seems to be the preferred approach in obstetric patients.

To our knowledge, only one randomized controlled trial involved the difference between the TAPB and QLB blocks in the management of acute postoperative pain [11]. In the study by Blanco et al., QLB was superior to TAPB in alleviating pain severity, as measured using the VAS. Moreover, patients in the QLB group consumed less morphine with patient-controlled analgesia (PCA). Contrarily, a recent meta-analysis of randomized controlled trials performed by El-Boghdadly et al. did not prove the superiority of one block over the other due to data inconsistency and methodological limitations. The authors suggested that both interventions provided comparable postoperative analgesia and opioid-sparing effects. Interestingly, both techniques were superior to inactive control only in the absence of intrathecal morphine [12]. Previously published randomized controlled studies presented the advantages of trunk blocks, such as QLB or TAPB, in comparison to standard care groups or sham blocks [15,20,21]. QLB topographically covers a broader field (T7-T12) in comparison to the ultrasound-guided TAPB (T10–T12) [22]. Ultrasound-guided or lateral TAPB has a high success rate and is safer compared with a landmark-based technique (a posterior approach through the triangle of Petit); however, it seems less effective in pain relief than the posterior approach [23]. Moreover, the lateral TAPB failed to relieve pain better than spinal morphine [24]. More studies are needed to present the advantages of different types of QLB regarding pain relief. However, the second type of QLB seems to be safer in comparison to QLB I due to a further distance from the perineum and visceral organs [25].

Although a statistical difference was found in the number of patients who received morphine, the total dose of opioids used did not differ between the groups. We suggest that this result was caused by the manner morphine was administered in the study and could be incidental. A subcutaneous route of morphine administration is controversial if other less invasive approaches, such as an oral route, are available. However, this manner was preferred by personnel of the maternity ward. The other explanation for higher morphine expenditures in the TAPB group could be a slightly lower consumption of tramadol among these patients in comparison to individuals after QLB (Table 5).

The total expenditure of intravenous opioids administered by a PCA pump is one of the best options to compare different analgesic technique. PCA possesses advantages, for example, in measuring the precise expenditure of opioids over time, drawing the drug–response curve and having excellent control of pain. However, as patients need to be immobilized by their connection to the pump, which is not coherent with the ERAS (enhanced recovery after surgery) protocol implemented in our Obstetric Department for elective cesarean sections, PCA was not used in our study [26]. Without long-lasting regional anesthesia techniques such as TAPB or QLB, the implementation of the ERAS protocol would be extremely difficult.

The main limitation of our study was the lack of a control group without any postoperative analgesic block. We did not add any opioid through the spine, which could have affected pain severity after the procedure. We did not test the spinal block area with the pinprick technique. The other drawback of our trial was associated with the route of administration of morphine and the lack of patient-controlled analgesia techniques, which could enable a better comparison with other randomized controlled trials.

## 5. Conclusions

In our study, none of the evaluated regional blocks demonstrated an advantage over the other in regard to acute postoperative pain management.

## Figures and Tables

**Table 1:** Patient demographic.

Group	TAPB (*n* = 93)	QLB (*n* = 94)	*p*-Value
Age (years)	31.48 (30.59–32.37)	32.55 (31.63–33.47)	0.67
Weight (kg)	80.39 (78.08–82.71)	78.52 (76.47–80.57)	0.71
Height (m)	1.67 (1.66–1.68)	1.66 (1.65–1.68)	0.91
BMI (kg/m^2^)	28.95 (28.17–29.74)	28.64 (27.91–29.37)	0.88

Legend: Results are presented as means and 95% confidence intervals. TAPB—transversus abdominis plane block; QLB—quadratus lumborum block.

**Table 2:** Indications for cesarean delivery.

Indication	Group		*p*-Value
	TAPB (*n* = 93)	QLB (*n* = 94)	
Subsequent cesarean section	45	53	0.27
Fetal malpresentation	21	25	0.52
Nonreassuring fetal heart rate tracing	9	7	0.59
Failure to progress	8	5	0.38
Others	10	4	0.09

Legend: TAPB—transversus abdominis plane block; QLB—quadratus lumborum block.

**Table 3:** Postoperative pain severity during the first 24 after the surgery.

	Mean VAS Results (CI)		
Time After Surgery in Hours	TAPB	QLB	*p*-Value
2	34.47 (29.26–39.69)	33.20 (28.18–38.61)	0.73
4	38.86 (34.20–43.53)	42.42 (37.75–47.08)	0.28
8	39.54 (35.14–43.93)	43.67 (39.27–48.06)	0.20
12	35.14 (30.78–39.50)	39.12 (34.76–43.48)	0.20
24	32.25 (27.67–36.83)	35.91 (31.34–40.49)	0.29
Mean VAS	36.05 (32.94–39.17)	38.90 (35.79–42.02)	0.64

Legend: VAS results are presented as means and confidence intervals. A two-sided *t*-test was incorporated for comparison of VAS results at consecutive hours and ANOVA for the mean VAS values (from 5 measurements). TAPB—transversus abdominis plane block; QLB—quadratus lumborum block.

**Table 4:** Mean analgesic drug consumption during the first 24 h after the surgery.

Antinociceptive Drug	Type of Regional Block		*p*-Value
	TAPB	QLB	
Ketoprofen	107.45 (91.21–123.69) mg	102.22 (84.47–119.98) mg	0.59
Diclofenac	22.34 (13.96–30.71) mg	15.00 (6.45–23.56) mg	0.45
Paracetamol	1117.02 (911.42–1322.62) mg	1044.44 (853.69–1235.20) mg	0.80
Tramadol	21.28 (11.96–30.59) mg	28.89 (16.33–41.45) mg	0.48
Morphine	4.02 (3.28–4.76) mg	3.09 (2.36–3.83) mg	0.085

Legend: The data are presented as mean doses of nonsteroidal anti-inflammatory drugs (NSAIDs), tramadol and morphine in milligrams, administered in both study groups on patients’ demand during the first postoperative day. Data presented in the table as the mean doses of drugs in milligrams and confidence intervals are given per patient in each of the study groups. Probability was calculated with the Mann–Whitney U test. TAPB—transversus abdominis plane block; QLB—quadratus lumborum block, mg—milligrams.

**Table 5:** Postoperative pain severity during the first 24 h after the surgery according to indications for cesarean section and type of regional analgesia.

Indication	Type of Regional Block		*p*-Value
	TAPB	QLB	
Subsequent cesarean section	34.74 (30.18–39.30)	39.08 (34.98–43.19)	0.16
Fetal malpresentation	42.47 (34.75–50.19)	37.01 (29.64–44.37)	0.31
Nonreassuring fetal heart rate tracing	32.13 (24.23–40.04)	34.11 (25.15–43.08)	0.73
Failure to progress	30.04 (15.31–44.77)	42.24 (27.51–56.97)	0.21
Others	35.64 (27.87–43.41)	47.07 (34.38–59.75)	0.12

Legend: VAS results from subsequent five measurements of pain intensity presented in millimeters as means and confidence intervals (CI). *p*-values were calculated with repeated measurements ANOVA. TAPB—transversus abdominis plane block; QLB—quadratus lumborum block.

## Data Availability

Due to the applicable privacy regulation (GDPR) and Good Clinical Practices (GCP) legislation, the full underlying dataset supporting the study cannot be provided. Anonymized data is available on motivated request and can be send to corresponding author—Michał Borys (michalborys1@gmail.com).

## Abbreviations

ANOVA: analysis of variance; CI: confidence interval, CPSP: chronic postsurgical pain; ERAS: enhanced recovery after surgery; NPSI: Neuropathic Pain Symptom Inventory; PCA: patient-controlled analgesia; TAPB: transversus abdominis plane block; VAS: visual analogue scale; QLB: quadratus lumborum block.
